# Assessing the impact of intervention strategies on dengue dynamics in Shenzhen, China

**DOI:** 10.1371/journal.pntd.0014521

**Published:** 2026-08-05

**Authors:** Qi Tan, Jia Wan, Cong Niu, Wei Liu, Dongfeng Kong, Zhen Zhang, Yuan Bai, Zhanwei Du

**Affiliations:** 1 College of Computer and Information Engineering and College of Artificial Intelligence, Nanjing Tech University, Nanjing, China; 2 Shenzhen Center for Disease Control and Prevention, Shenzhen, China; 3 West China School of Public Health and West China Fourth Hospital, Sichuan University, Chengdu, China; 4 School of Public Health and Emergency Management, Southern University of Science and Technology, Shenzhen, China; Sichuan University, CHINA

## Abstract

**Background:**

A dramatic increase in dengue infections has been observed in recent years, raising concerns regarding the potential further spread of dengue. Shenzhen, a major international port city in China, is typically a non-endemic region; however, it faces persistent risks from imported cases. The resurgence of imported risk has made the identification of effective prevention and control strategies a pressing public health priority for the region.

**Methodology:**

We integrated epidemic, environmental, and intervention data from Shenzhen covering the period from 2015 to 2023. A compartmental mathematical model was developed to characterize the transmission dynamics of dengue triggered by imported cases. We applied sensitivity analysis and developed a quantitative metric to evaluate the relative efficacy of various non-pharmaceutical intervention strategies in this specific urban context.

**Findings:**

Our sensitivity analysis identified vector control and the reduction of mosquito biting rates as the most critical factors influencing transmission dynamics. These analytical results were further validated through simulations based on a model fitted to historical data, which revealed that prioritizing these strategies significantly mitigated dengue transmission risks. Compared to alternative measures, these targeted interventions demonstrated a substantially higher impact on reducing the risk and scale of local outbreaks.

**Conclusions:**

This study provides a practical, evidence-based tool for health authorities to prioritize interventions in at-risk hub cities. Our findings underscore that for non-endemic port cities like Shenzhen, focusing on rigorous vector management and biting rate reduction is critical for mitigating the risk of dengue epidemics. These insights offer a strategic framework for guiding future epidemic control efforts against vector-borne diseases in non-endemic urban environments.

## Author summary

Dengue infections have increased dramatically in recent years; furthermore, fuelled by climate change, the spatial range suitable for dengue epidemics may expand. Non-pharmaceutical interventions to control the spread of the dengue virus are therefore critical. In this study, the authors developed mathematical models to characterize the dynamics of export-triggered dengue epidemics and, based on this, quantitatively analyzed the effects of various intervention strategies. The authors focused on Shenzhen, an international port city in China, which is at risk of imported infections, and revealed that the most effective measures for containing dengue in Shenzhen include vector control and reduced biting rates. This study provides a comprehensive and practical tool to support evidence-based intervention decision-making in containing the ongoing spread of dengue infections.

## 1. Introduction

Dengue is a mosquito-borne flavivirus disease that has caused over 14.1 million infection cases of dengue globally in 2024 [1]. In the Region of the Americas alone, cases have exceeded seven million by the end of April 2024, far surpassing the previous annual peak of 4.6 million in 2023 [2]. This marks a threefold increase compared to the same period last year, highlighting the urgent nature of this public health crisis. In the South-East Asia Region, many member states have environmental conditions that favor endemic dengue transmission [3,4]. Indonesia, in particular, is experiencing a significant rise in dengue cases, reporting 88,593 confirmed infections and 621 fatalities as of April 30, 2024, approximately three times the number of cases reported during the same period in 2023. This alarming trend underscores growing concerns regarding the escalating risk of dengue epidemics and their potential spread to new regions [5].

The dengue virus infection is heavily influenced by climate conditions [6–8]. The climate conditions limit the transmission of dengue via its effect on the vector life cycle [9]. However, due to climate change, there are increased precipitation, humidity, and rising temperatures in many regions, all of which create favorable conditions for the reproduction of mosquito vectors and the transmission of the virus [10–12]. Additionally, the movement of infected individuals and goods that may harbor mosquito vectors significantly influences the invasion patterns of dengue [13,14]. Regional spread is shaped by a combination of mobility, climate conditions, population density and control measures [15,16]. To prevent the widespread emergence of dengue, it is essential to implement effective interventions in at-risk hub areas [17]. Strategies should focus on vector control, border health measures, and improved clinical management to mitigate these risk factors [18,19].

**Fig 1:**
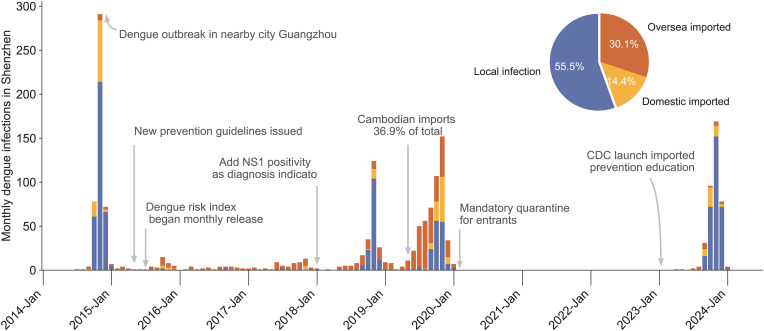
Dengue infection in Shenzhen (2014-2023). In 2014, a major dengue outbreak in Guangzhou, Guangdong Province, significantly affected neighboring cities, including Shenzhen. Subsequently, new guidelines for dengue prevention and control were implemented in Guangdong Province in 2015 and remain in effect. Data for 2020–2022 are omitted from the figure as only a few imported cases were detected and no local transmission was reported during this period. In 2023, following the relaxation of COVID-19 restrictions, dengue infections experienced a resurgence, primarily driven by an increase in imported cases.

Shenzhen, an international mega city in Guangdong Province, southern China, is typically a non-endemic region for dengue but has faced challenges from imported cases. Fig 1 shows a fluctuating dynamic pattern of dengue infections from 2014 to 2023. Detailed annual data on the total annual dengue infections in Shenzhen throughout the study period are presented in Fig A in S1 Text. Between 2020 and 2022, the number of imported dengue infections significantly decreased due to COVID-19 prevention and control measures, with zero local infections. However, in 2014, Guangdong Province experienced its worst recorded dengue epidemic, driven by a combination of factors such as imported cases, high mosquito density, and favorable temperatures [20]. Following the new prevention guidelines for dengue fever prevention and control in Guangdong Province issued in 2015, health authorities implemented different measures to contain potential dengue outbreaks. In 2023, as COVID-19 restrictions were relaxed, dengue infections began to rise again, prompted by a resurgence of imported cases [21]. The reemergence of infections underscores the urgent need for evidence-based intervention planning to effectively manage the risk of dengue in Shenzhen. Moreover, the population movement is also an important factor for dengue virus diffusion [22,23]. Thus, the control of dengue in central and portal city is essential for containment of the introduction and spread of dengue [24].

We hypothesize that intervention strategies targeting vector control, especially those reducing mosquito biting rate, have a substantial impact on transmission dynamics and can effectively reduce the risk of dengue outbreaks. In this context, the documented interventions and epidemic data from Shenzhen present a valuable opportunity to analyze the impact of these measures on dengue transmission linked to imported cases. Critical questions arise for local health authorities regarding which measures to prioritize and the timing of their implementation. In this study, we collect and analyze epidemic, environmental, and intervention data from Shenzhen. We develop a compartmental model and create a quantitative metric based on sensitivity analysis to evaluate the efficacy of various intervention strategies. This approach enables us to gain insights into the effects of these strategies within Shenzhen’s unique environmental and risk factors. We validate our analytical findings through simulations. Our results indicate that vector control and reducing biting rates are the most effective strategy in Shenzhen. This study offers new insights into intervention assessment and planning for dengue containment in the context of imported infection risks, contributing to future epidemic control efforts.

## 2. Methods

### 2.1. Ethics statement

Data for this research were sourced from open-access databases, media reports, and existing literature, with results derived from systematic mathematical simulations. The Medical Ethics Committee of Nanjing Tech University granted an exemption for ethical approval and waived the need for informed consent. This decision was based on several factors: (1) the analysis utilized strictly de-identified data; (2) the protocol involved no biological specimens or direct medical interventions; and (3) the research outcomes had no impact on the clinical treatment or management of patients.

### 2.2. Disease transmission model

This study builds upon the SEI-SEIR compartment model for dengue transmission among hosts and vectors proposed by Caldwell et al. [25] by integrating a Quarantine state. In the SEI-SEIR model, climate variables have been incorporated into the dynamics of both the vector and the epidemic. Additionally, the quarantine state was introduced to account for non-pharmaceutical interventions within the model’s framework. The formulation of the proposed SEI-SEIQR model is presented below:


$$\begin{array}{cc} \frac{dS_{v}}{dt} & =\alpha_{v}\frac{1}{\mu_{v}}N_{v}(1-\frac{N_{v}}{K})-b\beta_{v}\frac{\left(I_{h}+p_{i}Q_{h}\right)}{N_{h}}S_{v}-\mu_{v}*S_{v}\\ \frac{dE_{v}}{dt} & =b\beta_{v}\frac{\left(I_{h}+p_{i}Q_{h}\right)}{N_{h}}*S_{v}-(\gamma_{v}+\mu_{v})*E_{v}\\ \frac{dI_{v}}{dt} & =\gamma_{v}*E_{v}-\mu_{v}*I_{v}\\ \frac{dS_{h}}{dt} & =(\mu_{n}+ie)*N_{h}-\frac{b\beta_{h}I_{v}}{N_{h}}*S_{h}-(\mu_{h}+ie)*S_{h}\\ \frac{dE_{h}}{dt} & =\frac{b\beta_{h}I_{v}}{N_{h}}*S_{h}-(\gamma_{h}+\mu_{h}+ie)*E_{h}\\ \frac{dI_{h}}{dt} & =\gamma_{h}*E_{h}-((1-p_{q})\sigma_{h}+p_{q}\sigma_{q}+\mu_{h}+ie)*I_{h}\\ \frac{dQ_{h}}{dt} & =p_{q}\sigma_{q}*I_{h}-(\theta_{h}+\mu_{h}+ie)*Q_{h}\\ \frac{dR_{h}}{dt} & =(1-p_{q})\sigma_{h}*I_{h}+\theta_{h}*Q_{h}-(\mu_{h}+ie)*R_{h} \end{array}$$ (1)

where $\alpha_{v}=EFD*pEA*MDR$. The parameter descriptions are provided in Table 1. The compartment $S_{v}$, $E_{v}$ and $I_{v}$ represent the disease transitions of the vector, while $S_{h}$, $E_{h}$, $I_{h}$ and $R_{h}$ represent the disease transitions of humans. The value of several key epidemiological parameters, such as *b*, *EFD*, *pEA*, *MDR* and $\mu_{v}$, are temperature dependent. The temperature-dependent functional forms of these key vector trait coefficients are presented in Table A in S1 Text. The carrying capacity of vector is temperature and rainfall dependent. Following [25,31], the adult mosquito carrying capacity, *K*, is modeled as a function of temperature temperature (*T*) and accumulated 14-day rainfall *R*:


$$\begin{array}{c} K(T,H,R)=\frac{\text{EFD}(T_{0})\cdot \text{pEA}(T_{0})\cdot \text{MDR}(T_{0})\cdot \mu (T_{0}{)}^{-1}-\mu (T_{0})}{\text{EFD}(T_{0})\cdot \text{pEA}(T_{0})\cdot \text{MDR}(T_{0})\cdot \mu (T_{0}{)}^{-1}}\cdot N_{m,\max}\\ \cdot e^{\frac{-E_{A}\cdot (T-T_{0}{)}^{2}}{K_{B}\cdot (T+273)\cdot (T_{0}+273)}}\cdot f(R) \end{array}$$

where *T*_0_ represent the optimal temperature for mosquito abundance, $N_{m,\max}$ is the maximum possible mosquito abundance (scaled to the human population), and $E_{A}$ denotes the activation energy. The impact of rainfall on carrying capacity is captured by the function *f*(*R*) is modeled as $f(R_{\text{Briere}})=c\cdot R\cdot (R-R_{\min})\cdot \sqrt{\left(R_{\max}-R\right)}\cdot z.$ The climate-related parameters were parameterized as follows: $T_{0}=29^{\circ }\text{C}$, $N_{m,\max}=2*N_{h}$, $K_{B}=8.617\times 10^{-5}\text{ eV/K}$, activation energy $E_{A}=0.05\text{ eV}$, $c=7.86\times 10^{-5}$, $R_{\min}=1\text{mm}$, and $R_{\max}=123\text{mm}$.

**Table 1:** Table of parameters.

	Definition	Values	Sources
$\mu_{n}$	Human natural birth rate (per 1000 people)	10.6	Worldbank [26]
$\mu_{h}$	Human death rate (per 1000 people)	7.12	Worldbank [26]
*i.e.,*	Immigration/emigration rate	0.00014	Worldbank [27]
$1/\gamma_{h}$	Human incubation period (days)	5.9	[28]
$1/\sigma_{h}$	Human infectivity period (days)	5.0	[28]
$1/\sigma_{q}$	Detection interval of local infection (days)	Shenzhen: 4.2-4.7	Shenzhen CDC
$1/\theta_{h}$	Quarantine period (days)	Shenzhen: 1–2	Shenzhen CDC
$p_{i}$	Probability of Q state in infection	0	Shenzhen CDC
$p_{q}$	Detection probability	0.28	[29]
*b*	Biting rate	Temperature dependent	[30]
*EFD*	Eggs laid per female per day		
*pEA*	Probability of mosquito egg to adult survival		
*MDR*	Mosquito. egg to adult development rate (per day)		
$\mu_{v}$	Mortality rate of vector		
$\beta_{h}$	Vector to human transmission probability		
$\beta_{v}$	Human to vector transmission probability		
$1/\gamma_{v}$	Vector incubation period		
K	Carrying capacity	Temperature dependent and rainfall dependent	[25]
$z_{\xi }$	Localize coefficient of biting rate	Estimated	
$\bar{\xi }$	Decline of biting rate after risk map release	Estimated	
η	Policy due vector mortality increase	Estimated	

The parameter $p_{q}$ represents the probability of detection by the health department, which is influenced by factors such as medical-seeking behavior and the capacity of health surveillance. We set the model parameter $p_{q}$ using serological priors. We adopt a 28% symptomatic ratio from the 2023 Shenzhen outbreak survey [29], supported by regional data from Zhongshan (31%) [32]. These biological anchors reduce the degrees of freedom and minimize reliance on noisy clinical reports, ensuring more stable estimation of transmission parameters. The parameter $p_{i}$ serves as a reduction coefficient for the transmission probability from quarantined individuals ($Q_{h}$) to the susceptible vector population ($S_{v}$). Specifically, $p_{i}$ quantifies the effectiveness of clinical isolation measures, such as the use of mosquito-proof hospital wards and insecticidal treatments in medical facilities, in decreasing the contact rate between vectors and infected patients.

### 2.3. Basic reproduction number and sensitivity analysis

We employ the next generation matrix method [33] to calculate the basic reproduction number *R*_0_ based on the disease transmission model in Eq 1. The formulation of *R*_0_ can be expressed as follows.


$$\begin{array}{cc} R_{0} & =\frac{b^{2}\;\beta_{h}\;\beta_{v}\;\gamma_{h}\;\gamma_{v}\;\left(ie+\mu_{h}+\theta_{h}+p_{i}\;p_{q}\;\sigma_{q}\right)}{\mu_{v}\;\left(\gamma_{v}+\mu_{v}\right)\;\left(\gamma_{h}+ie+\mu_{h}\right)\;\left(ie+\mu_{h}+\theta_{h}\right)\;\left(ie+\mu_{h}+\sigma_{h}-p_{q}\;\sigma_{h}+p_{q}\;\sigma_{q}\right)}\frac{N_{v}^{*}}{N_{h}} \end{array}$$ (2)

where $\frac{N_{v}^{*}}{N_{h}}=m(1-\frac{\mu_{v}^{2}}{\alpha_{v}})$. Then the probability of a major outbreak is approximated by [34]:


$$\begin{array}{c} OP=1-(\frac{1}{R_{0}}{)}^{i}, \end{array}$$ (3)

where *i* represents the initial number of infectious individuals. To assess the influence of epidemiological parameters on disease transmission, we conduct a sensitivity analysis on key parameters, including vector mortality and the duration of quarantine. The normalized sensitivity index of *R*_0_ with respect to a parameter *x* is defined as [35]:


$$\begin{array}{c} SA(R_{0},x)=\frac{\partial R_{0}}{\partial x}\cdot \frac{x}{R_{0}}. \end{array}$$ (4)

This index measures the relative change in *R*_0_ with respect to the relative change in parameter *x*. Specifically, the sensitivity indices for the vector mortality rate $\mu_{v}$ and the $\theta_{h}$ are calculated as:


$$\begin{array}{c} SA(R_{0},\mu_{v})=\frac{\partial R_{0}}{\partial \mu_{v}}\cdot \frac{\mu_{v}}{R_{0}}\;\text{and}\;SA(R_{0},\theta_{h})=\frac{\partial R_{0}}{\partial \theta_{h}}\cdot \frac{\theta_{h}}{R_{0}}. \end{array}$$ (5)

Moreover, we can also calculate the sensitivity index with respective to outbreak probability. We derive the mathematical expression the different sensitivity index using Symbolic Math Toolbox in Matlab [36]. The sensitivity index can be interpreted as the percentage change in *R*_0_ resulting from 1% change in the epidemiological parameters [37]. Specifically, we concentrate on three key types of parameters associated with intervention measures: vector mortality ($\mu_{v}$) related to vector control, biting rate (*b*) associated with risk informing, and detection probability ($p_{q}$), detect interval ($\sigma_{q}$), and quarantine quality ($p_{i}$) linked to surveillance and clinical management.

### 2.4. Epidemiological parameter inference

The prioritization of interventions is contingent upon the specific conditions of each locality. To identify the most effective intervention measures, it is essential to access localized parameters. We utilize epidemic and environmental data to derive these localized parameters through Markov Chain Monte Carlo (MCMC) methods. Epidemic data document the sources of infection, distinguishing between local and imported cases. Imported cases are influenced by external factors; therefore, to assess the local epidemiological parameters, we enhance the model presented in Eq 1 by differentiating between these two infection states. Additionally, recognizing that real-world epidemic data is affected by local intervention strategies, such as vector control and the dissemination of risk maps, we incorporate the effects of these interventions into our parameter estimation process:


$$\begin{array}{cc} \frac{dS_{v}}{dt} & =\alpha_{v}N_{v}(1-\frac{N_{v}}{K})-b_{t}\beta_{v}(T)\frac{\left(I_{h}+{\hat{I}}_{h}+p_{i}Q_{h}\right)}{N_{h}}S_{v}-\mu_{v,t}*S_{v}\\ \frac{dE_{v}}{dt} & =b_{t}\beta_{v}(T)\frac{\left(I_{h}+{\hat{I}}_{h}+p_{i}Q_{h}\right)}{N_{h}}*S_{v}-(\gamma_{v}(T)+\mu_{v,t})*E_{v}\\ \frac{dI_{v}}{dt} & =\gamma_{v}(T)*E_{v}-\mu_{v,t}I_{v}\\ \frac{dS_{h}}{dt} & =\mu_{h}N_{h}-\frac{b_{t}\beta_{h}(T)I_{v}}{N_{h}}S_{h}-\mu_{h}S_{h}\\ \frac{dE_{h}}{dt} & =\frac{b_{t}\beta_{h}(T)I_{v}}{N_{h}}*S_{h}-(\gamma_{h}+\mu_{h}{)}^{*}E_{h}\\ \frac{dI_{h}}{dt} & =\gamma_{h}*E_{h}-((1-p_{q})\sigma_{h}+p_{q}\sigma_{q}+\mu_{h})*I_{h}\\ \frac{d{\hat{I}}_{h}}{dt} & =A_{t}^{*}-((1-p_{q})\sigma_{h}+p_{q}{\hat{\sigma }}_{h}+\mu_{h})*{\hat{I}}_{h}\\ \frac{dQ_{h}}{dt} & =p_{q}\sigma_{h}*I_{h}+p_{q}{\hat{\sigma }}_{h}*{\hat{I}}_{h}-(\theta_{h}+\mu_{h})*Q_{h}\\ \frac{dR_{h}}{dt} & =p_{h}\sigma_{h}I_{h}+\theta_{h}Q_{h}+p_{h}\sigma_{h}{\hat{I}}_{h}-\mu_{h}R_{h} \end{array}$$ (6)

where ${\hat{I}}_{h}$ denotes the imported infection with time varying force of infection $A_{t}^{*}$, where imported cases encompass both overseas and domestic sources. This equivalent treatment is consistent with empirical observations indicating that both types of imported cases exhibit highly comparable intervals from symptom onset to quarantine, thereby exerting a similar impact on local transmission dynamics. $b_{t}=\xi_{t}*b(T)$ and $\mu_{v,t}=\eta_{t}*\mu_{v}(T)$, where $\xi_{t}$ indicates the influence of intervention, e.g., release of risk map, on biting rate. We set $\xi_{t}=z_{\xi }$ before the risk map release and $\xi_{t}=z_{\xi }\bar{\xi }$ after the risk map release. Similarly, $\eta_{t}$ indicate the influence of intervention, e.g., vector control, on the vector mortality. We set $\eta_{t}=1$ before the vector control campaign and $\eta_{t}=\eta$ after the vector control campaign start.

The procedure of MCMC is outlined as follows. Let ${𝐱}_{t}=\{S_{v,t},E_{v,t},I_{v,t},S_{h,t},E_{h,t},I_{h,t},Q_{h,t},R_{h,t}\}$ denote the states of disease transmission model in Eq 6 at time slot *t* and θ denote the parameters *t*o be estimated. The epidemic data records the number of daily new detected infections, i.e., $p_{q}\sigma_{h}*I_{h}$ and $p_{q}{\hat{\sigma }}_{h}*{\hat{I}}_{h}$. As the epidemic is trigger by the imported infection, we use reported imported cases data to approximate the force of imported infection $A_{t}^{*}$ and use the local infection data as model observation for parameter inference. The likelihood of observing number of daily new detected infections $y_{t}$ is $L(y_{t}|{𝐱}_{t})=\mathbb{N}(y_{t}|p_{q}\sigma_{h}*I_{h},\sigma_{l})$. We execute the MCMC methods for a total of 50,000 steps, with an initial burn-in period of 10,000 steps.

## 3. Results

### 3.1. Climate condition and dengue infection in Shenzhen

**Fig 2:**
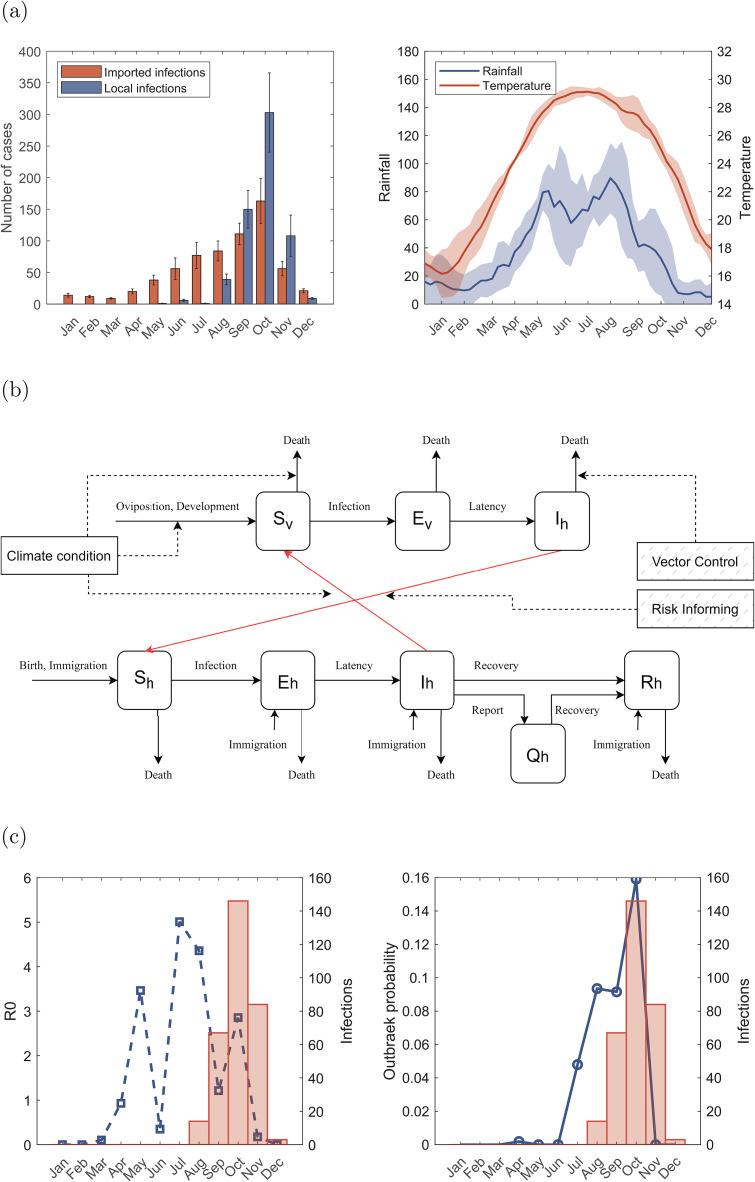
(a) Average number of imported and local infections (left) and average temperature & rainfall (right) in Shenzhen. (b) Illustration of the mathematical epidemic model. (c) Evaluated *R*_0_ with only considering the climate condition and the evaluated outbreak probability with only considering the climate condition imported infection in 2023. The red bar indicated the numbers of recorded infection cases.

The average infection and environmental data of Shenzhen are presented in Fig 2a. Imported infections persist consistently throughout the year, peaking notably in September and October. In contrast, local infections are also peak during September and October but less common in other months. Shenzhen is situated in a subtropical monsoon climate characterized by extended summers and brief winters. The region enjoys a temperate climate with abundant sunshine and rainfall. The annual average temperature is around 23.3°C. January marks the lowest average temperature, while July sees the peak at around 29.0°C. Annual precipitation averages at 1932.9 millimeters, with the majority of rainfall concentrated during the flood season from April to September.

### 3.2. Basic reproduction number and outbreak probability in Shenzhen

Considering the force of import infection and environmental condition, we use a mathematical dengue model to model the dynamic of local dengue infection (as illustrated in Fig 1b and the mathematical Eq is shown in Eq 1). Fig 1c showcases the computed basic reproduction number and the probability of outbreak triggered by the imported infections in 2023. The results of other years can be referred to supplementary information (Figs B and C in S1 Text). Outbreak probability demonstrated significantly stronger associations with reported cases (Pearson *r* = 0.485, *p* < 0.001; Spearman ρ=0.322,p=0.006) than *R*_0_, which showed weaker correlations (Pearson *r* = 0.091, *p* = 0.443; Spearman ρ=0.158,p=0.185). We observed that climate-based outbreak probability typically leads infection events by several months. The delay of local outbreak might caused by the intervention of local CDC. Therefore, we further introduce the intervention component in the mathematical model and inferred the effect of intervention.

### 3.3. Epidemiological parameter inference

The epidemiological intervention parameters are inferred from the Shenzhen epidemic data, along with environmental and intervention data. Birth rates, death rates, and immigration rates are based on national data for China, sourced from the World Bank. When considering the average values of $\bar{\xi }$ and η, the model inadequately captures the observed data for 2018 and 2023, indicating underfitting. Consequently, we proceed to independently derive intervention-specific parameters for the data from 2018 and 2023.

The mean estimations of three key epidemiological parameters, along with their 95% confidence intervals (CIs), are as follows: the localized biting scale is $z_{\xi }=1.40$ (95% CI: [1.10, 2.08]), the decline in biting rate due to information dissemination is $\bar{\xi }=0.63$ (95% CI:[0.34,0.94]), and the increase in vector mortality attributable to policy measures is η=1.70 (95% CI: [1.03, 2.64]). In Shenzhen, the biting rate aligns more closely with the temperate-driven function, with the localized biting scale $z_{\xi }$ estimated at approximately 140%. Environmental factors significantly influence dengue infections, with intervention measures playing a crucial role in shaping transmission dynamics. Risk communication leads to a nearly 40% reduction in the biting rate, while vector control efforts enhance vector mortality by 70%. The mean estimations year-specific epidemiological parameters and their 95% CIs are: ${\bar{\xi }}_{2018}=0.87$ (95% CI: [0.59, 0.99]), $\eta_{2018}=2.18$ (95% CI: [1.43, 3.54]), ${\bar{\xi }}_{2023}=0.79$ (95% CI: [0.51, 0.99]), $\eta_{2023}=1.59$ (95% CI: [1.02, 2.78]). When comparing these parameters across different years, the observed declines in biting rate and increases in vector mortality are relatively minor. This trend contributes to a higher probability of local dengue transmission. We will utilize the localized density of epidemiological parameters for the analysis and simulation of intervention effects. Figs D and E in S1 Text present the posterior probability density distributions of key epidemiological parameters and the fitting results of MCMC calibration, respectively.

### 3.4. Intervention priority using sensitivity analysis

The sensitivity analysis related to critical intervention targets for *R*_0_ and outbreak probability is illustrated in Table 2. This analysis indicates that the most effective intervention measures are biting rate control and vector control, with quarantine duration and detection probability ranking next in effectiveness. The simulation study by Claypool [19] reveals that in Colombia the most preferred measures for controlling dengue and chikungunya are insecticides and long-lasting insecticide-treated nets. These two interventions focus on vector control and biting reduction, respectively, which aligns with our findings. The full table can be refer to Table B in S1 Text. In the timing of intervention implementation, the most effective period occurs during September, October, and November, when imported infection rates and local transmission potential are elevated. Furthermore, May and July also merit significant attention.

**Table 2:** Sensitivity analysis results for key epidemiological parameters in Shenzhen. The amplitude of the sensitivity index indicates the strength of impact. Vector mortality and biting rates are the most influential key parameters for containing a dengue outbreak.

Parameter	Vector mortality $\mu_{v}$	Detect rate $1/\sigma_{q}$	Prop. of being quarantined $p_{q}$	Quarantine quality $p_{i}$	Biting rate *b*
2015	-0.045	-0.017	-0.0097	0.0001	0.0768
2016	-0.045	-0.017	-0.0097	0.0001	0.0768
2017	-0.045	-0.017	-0.0097	0.0001	0.0768
2018	-0.021	-0.009	-0.0048	0.0000	0.0377
2019	-0.045	-0.017	-0.0097	0.0001	0.0768
2023	-0.036	-0.014	-0.0078	0.0001	0.0617
Average	-0.040	-0.0154	-0.0086	0.0001	0.0678

**Fig 3:**
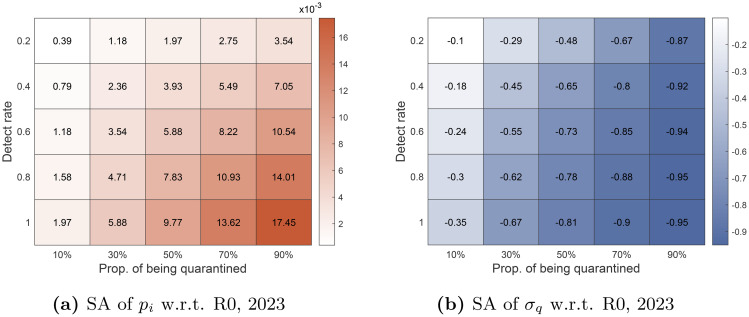
Sensitivity analysis of dengue infection outcomes under varying strengths of clinical management.

We further assess the effectiveness of different detection intervals and detection probabilities. Fig 3 illustrates the sensitivity index for the detection interval $\sigma_{p}$ and quarantine quality $p_{i}$. A high detection probability combined with a shorter interval leads to an increase in quarantine size, highlighting the importance of quarantine quality. Moreover, the importance of minimizing the detection interval becomes crucial, when detection probability is high,.

### 3.5. Simulation validation

**Fig 4:**
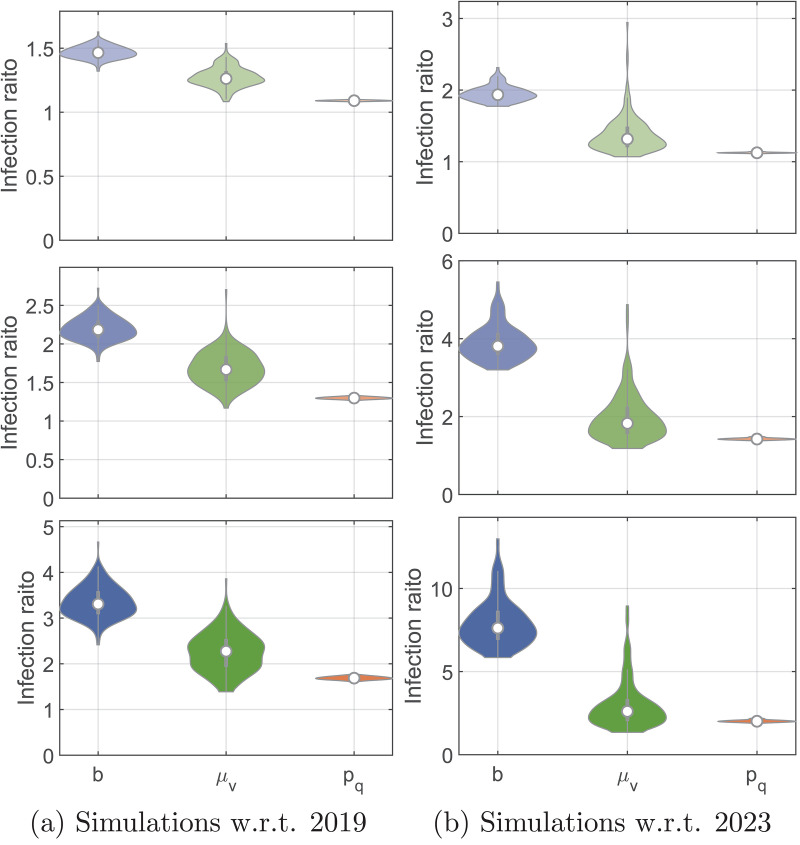
Simulation results of varying intervention strengths: The first row shows the effects of a 5% adjustment to a specific parameter, reflected in the infection ratio. The second and third rows present the outcomes for 10% and 15% adjustments, respectively.

We conducted simulations to assess the analytical effects of intervention measures by creating synthetic scenarios that vary key epidemiological parameters. For consistency in comparison, these parameters were adjusted to increase risk: specifically, we increased *b* while decreasing $\mu_{v}$ and $p_{q}$. The infection ratio was calculated as the total number of infections in the synthetic scenarios relative to that in the base scenario. The results are presented in Fig 4. The biting rate and vector mortality emerged as the most critical targets. A 15% increase in biting rate resulted in a threefold increase in the infection ratio for the 2019 setting and a sevenfold increase for the 2023 setting. A 15% change in vector mortality led to a nearly twofold increase in the infection ratio for both the 2019 and 2023 settings. These results align with the analytical results. Sensitivity simulation results for variable biting rates, vector mortality rates, and quarantine probabilities regarding annual epidemic simulations are presented in Figs F, G and H in S1 Text, respectively.

To inform practical policy-making, we evaluated the combinatorial effects of different intervention measures. We simulated scenarios where pairs of key parameters ($b,\mu ,p_{q}$) were simultaneously adjusted by ±5%. As shown in Table 3, the joint implementation of biting rate reduction (*b*) and vector mortality enhancement (μ) proved to be the most effective strategy, reducing the total infection scale by an average of 41.4%. This combined approach outperformed other pairings, such as $p_{q}+b$ and $p_{q}+\mu$, indicating that integrated vector management (targeting both the human-vector interface and population density) provides a more robust defense than single-target strategies. These simulation and analytical results support our hypothesis that intervention strategies targeting vector-related parameters can substantially influence transmission dynamics. In particular, the biting rate and vector mortality emerge as dominant drivers of outbreak magnitude, as relatively small perturbations in these parameters lead to disproportionately large increases in infection burden.

**Table 3:** Combinatorial effects of combined intervention measures (2015—2023). Values represent the ratio of total infections relative to the baseline scenario.

		2015	2016	2017	2018	2019	2023	Average
Weaken 5%	b+μ	1.726	1.920	1.543	2.418	1.723	2.270	1.933
	$p_{q}+\mu$	1.272	1.394	1.295	1.596	1.351	1.531	1.406
	$p_{q}+b$	1.601	1.628	1.347	1.803	1.474	1.752	1.601
Strengthen 5%	b+μ	0.629	0.564	0.711	0.463	0.625	0.523	0.586
	$p_{q}+\mu$	0.812	0.731	0.798	0.640	0.752	0.699	0.739
	$p_{q}+b$	0.649	0.637	0.765	0.579	0.700	0.608	0.656

## 4. Discussion

The burden of dengue on health and economy has increased in the recent years [2,3,38,39]. Numerous studies have focused on analyzing and projecting the impact of climate change on dengue transmission [31,40], enhancing our understanding of future risks associated with the disease. Most findings indicate that dengue risk is expected to worsen in the near future, particularly in temperate regions, where global warming is likely to create more favorable conditions for outbreaks [41,42]. Given these alarming trends, it is crucial to further investigate the effectiveness of existing control measures and implement appropriate interventions. Notably, mosquito-borne diseases are not limited to dengue. Guangdong Province, China, has recently experienced a large-scale chikungunya outbreak. Following the identification of an index chikungunya case on July 8, 2025, a significant epidemic emerged in Foshan, Guangdong. Between June 16 and July 31, 2025, reported cumulative cases reached 2,658 [43]. China documented its first imported case of chikungunya fever in 2008 and local outbreaks linked to imported cases were recorded in Guangdong, Zhejiang, and Yunnan provinces between 2010 and 2019 [44]. Therefore, our modeling framework offers a scalable foundation for managing other mosquito-borne diseases triggered by imported infections, such as chikungunya. Because dengue and chikungunya share the same *Aedes* vector, vector-intrinsic parameters, including carrying capacity, natural mortality rate, and biting rate, are largely transferable across models. The parameters for the SEI-SEIQR model were categorized into shared and disease-specific groups, as detailed in Table C in S1 Text. Notably, host- and vector-related parameters are considered transferable between diseases with similar dynamics, such as dengue and chikungunya. However, the structural SEI-SEIQR framework requires recalibration of virus-specific parameters, such as transmission probability and the duration of infectivity, to account for the unique biological characteristics of the chikungunya virus. While the modeling approach for evaluating intervention efficacy remains consistent, location-specific environmental factors (e.g., temperature) and disease-specific dynamics necessitate a tailored parameterization to ensure accurate local forecasting.

In this study, we propose an analytical framework to assess the impact of control measures and enhance our understanding of policy priorities. Concerns about the expansion of dengue infection regions are heightened by climate change. We conducted an empirical study in Shenzhen, an international port city in China, taking into account the influence of imported dengue cases. Shenzhen experiences significant cross-border interactions with dengue-endemic regions, such as South Asian countries, and local dengue infections in 2023 reached their highest levels since 2015. Our findings indicate that a decline in biting rates and effective vector control are the most critical measures for reducing dengue transmission. We validated these analytical results through simulations. As Shenzhen is a non-endemic region where local infections are primarily triggered by imported cases, our analysis suggests that interventions would be more effective when the influx of imported infections is high. Overall, our study highlights the importance of targeted interventions in relation to imported dengue cases. To measure the force of infection, we use historical data on imported infections. For future projections and planning, it is essential to consider that external epidemic environments fluctuate annually. Therefore, incorporating transportation data alongside infection status in endemic regions will provide a more comprehensive understanding of the risks associated with imported infections. Given the progress in dengue vaccines [45,46], recommending international travelers to get vaccinated would be a good option in the future.

Evaluating interventions is crucial for effective disease prevention. Simulation-based studies are often employed to assess the effectiveness of various intervention strategies, with a particular focus on vector control and the reduction of biting rates. Our study advances this field by proposing an analytical framework to identify critical intervention periods. Sensitivity analysis is commonly used to evaluate how changes in parameters influence model outputs, thereby identifying the most impactful factors for guiding intervention policies [37,47,48]. In this study, we utilize the sensitivity index due to its computational efficiency and its ability to analyze effects over time. Temporal and geographical heterogeneity significantly influence epidemic dynamics, thereby affecting the effectiveness of interventions. To support adaptive intervention planning, real-time surveillance data (e.g., vector density and imported cases) can be fed into the model to continuously update risk estimates. When the probability of an outbreak exceeds a specific threshold, it triggers immediate mid-season adjustments to the intervention strategy, ensuring the most efficient use of public health resources. To address the inherent challenges in estimating the detection probability from passive surveillance data, we constrained this parameter using empirical serological evidence. We informed the model’s prior for detection probability based on the symptomatic ratio of dengue infections. Sensitivity analysis shows that while detection probability affects the predicted infection magnitude, our conclusions on intervention timing remain robust. Integrating local and regional serological data mitigates the low signal issue in sparse data years, ensuring reliable model outputs for public health planning.

The current study has several limitations. First, regarding parameter inference, our data only include information on the initiation time of interventions. This restricts our ability to evaluate the varying impacts of additional measures implemented during the intervention period, leading our assessment to focus primarily on the average effects of the interventions. Future research could enhance the fidelity of these simulations by incorporating time-varying efficacy functions, such as exponential decay or piecewise functions, to reflect the potential waning of public adherence or the diminishing returns of vector control operations. Integrating granular operational data, such as daily insecticide usage or public engagement metrics with health advisories, would allow for a more nuanced assessment of how fluctuating intervention strength influences epidemic trajectories. Second, key vector-related parameters are modeled as temperature-dependent functions. These functional forms are adopted from existing study that integrates the several laboratory studies. However, potential discrepancies in local ecological or epidemiological conditions may introduce bias into parameter specification. Third, we employ a compartmental model at the city-wide level. This approach implicitly assumes homogeneous mixing and averages over spatial heterogeneity within Shenzhen, such as differences between urban and rural areas. As a result, localized transmission dynamics and region-specific intervention effects are not explicitly captured. Last, while we can conduct a cost-effectiveness analysis based on the effectiveness findings, the costs of interventions involve complex factors, including labor, human resources, and materials. At this stage, we lack the necessary data to pursue this analysis further.

In conclusion, the risk of dengue has markedly increased, with climate change significantly influencing transmission dynamics. Our study underscores the urgent need to evaluate the effectiveness of existing control measures, particularly in light of the projected worsening of dengue risk. By proposing an analytical framework to assess intervention impacts, we provide valuable insights into policy priorities for dengue prevention. The empirical study conducted in Shenzhen highlights the importance of effective vector control and reduced biting rates, particularly in a non-endemic region where local infections are primarily driven by imported cases. Our findings indicate that interventions are more effective during periods of high imported infections, emphasizing the need for targeted strategies. Despite the limitations in parameter inference and the complexity of cost-effectiveness analysis, our research contributes to a deeper understanding of dengue dynamics and the critical role of effective detection in mitigating local transmission.

## Supporting information

S1 Text Supplementary information of assessing the impact of intervention strategies on dengue dynamics in Shenzhen, China. (PDF)
