# Supplementary material for: Assessing the impact of intervention strategies on dengue dynamics in Shenzhen, China
Source: PLoS Negl Trop Dis. 2026 Aug 5;20(8):e0014521. doi: 10.1371/journal.pntd.0014521 (PMC13472445; doi:10.1371/journal.pntd.0014521)
Supplement: Supplementary file 1 [file pntd.0014521.s001.pdf]

Supplementary Information of Assessing the Impact of Intervention Strategies on Dengue Dynamics in Shenzhen, China

List of Figures

A Annual dengue infection cases in Shenzhen 2
B Temporal patterns of R_0 and outbreak probability (2015-2017) 3
C Temporal patterns of R_0 and outbreak probability (2018-2019, 2023) . 4
D Posterior probability density distributions for key epidemiological parameters estimated by MCMC 5
E Fitting results of the MCMC calibration. 6
F Simulated epidemic outcomes under varying biting rates across years. 7
G Simulated epidemic outcomes under varying vector mortality rates across years. 8
H Simulated epidemic outcomes under varying quarantine probabilities across years 8

List of Tables

A Temperature-dependent functional forms of the key coefficients for vector traits. 9
B Monthly sensitivity index of key epidemiological parameters. 10
C Classification of shared and disease-specific parameters for the SEI-SEIQR model 11

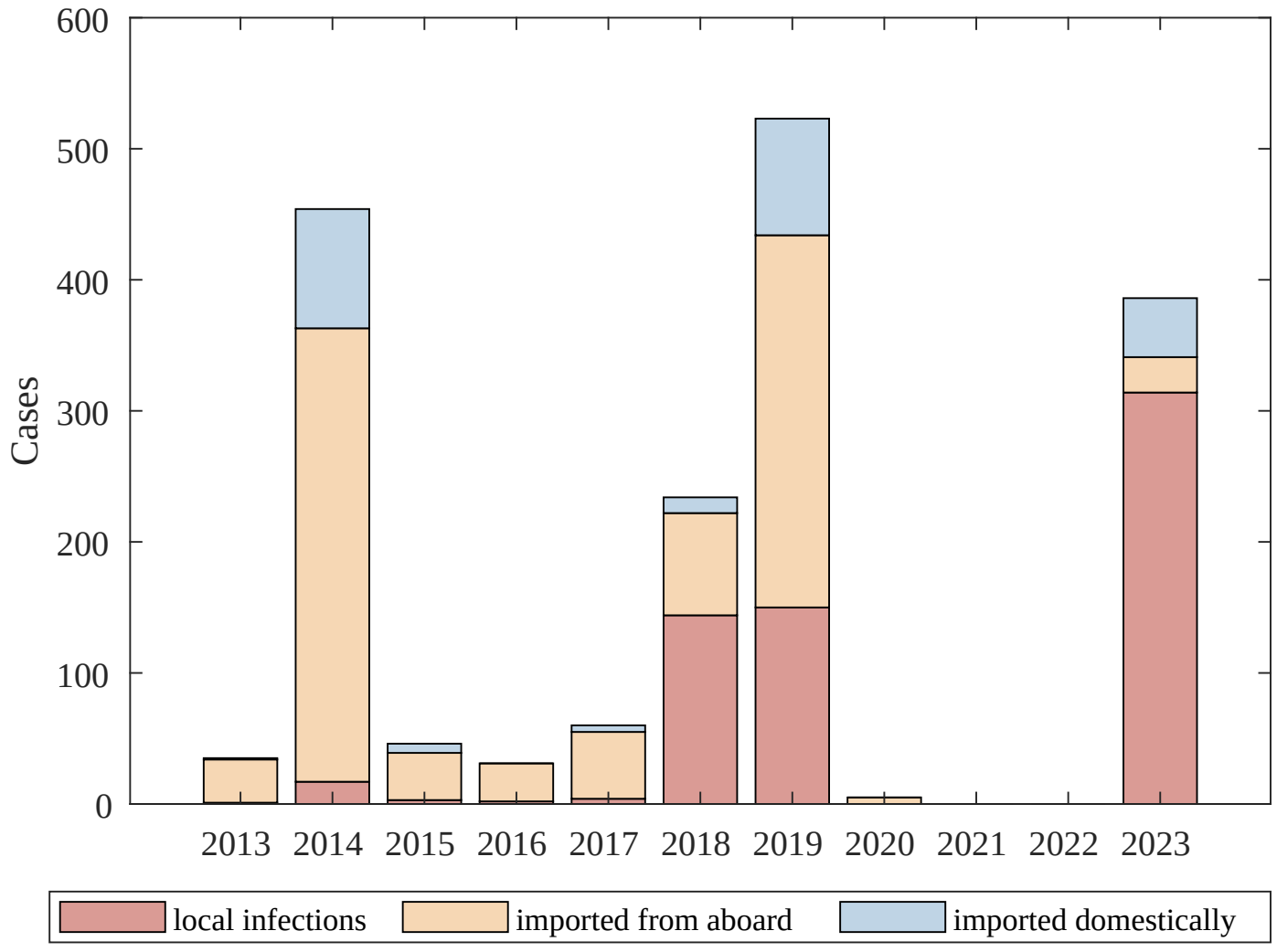

Fig A: **Annual dengue infection cases in Shenzhen**. No reported cases were recorded during 2020–2022 due to COVID-19 intervention measures, and thus these years were excluded from subsequent analysis.

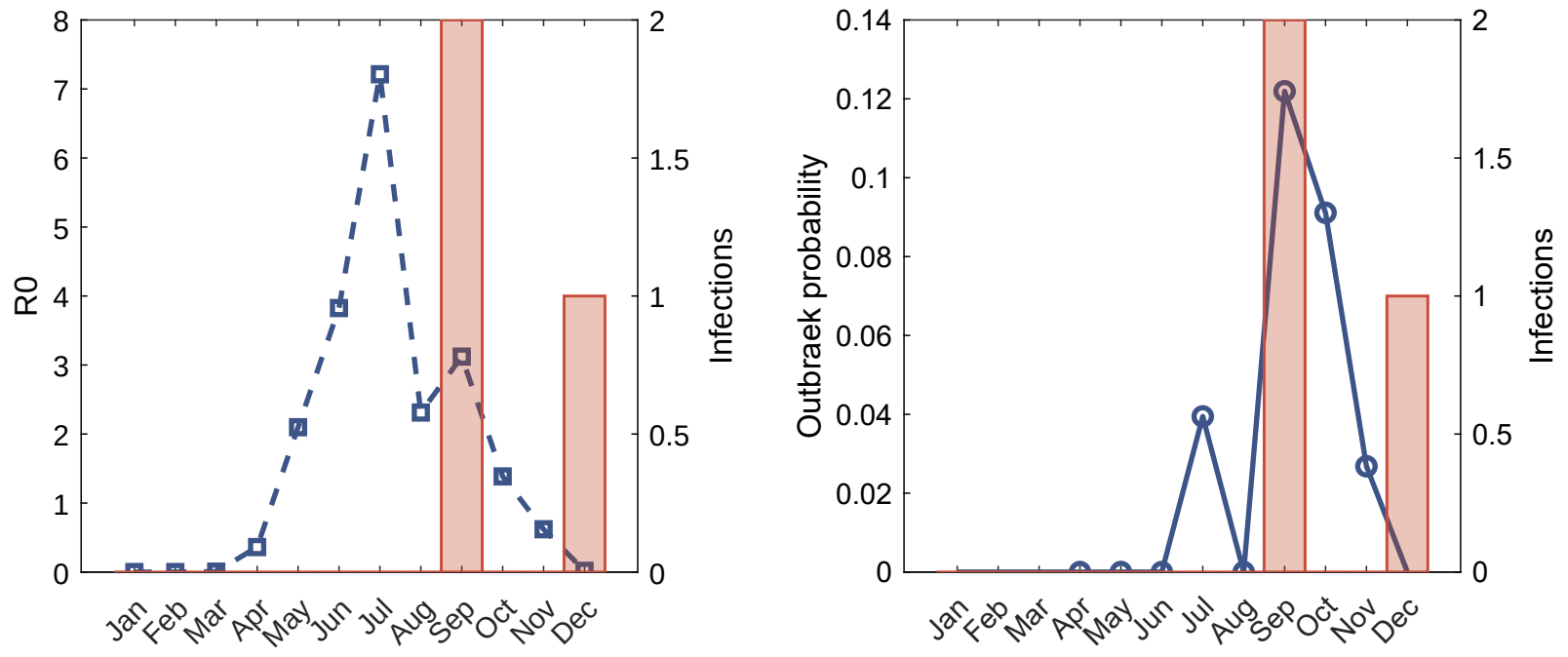

(a) 2015

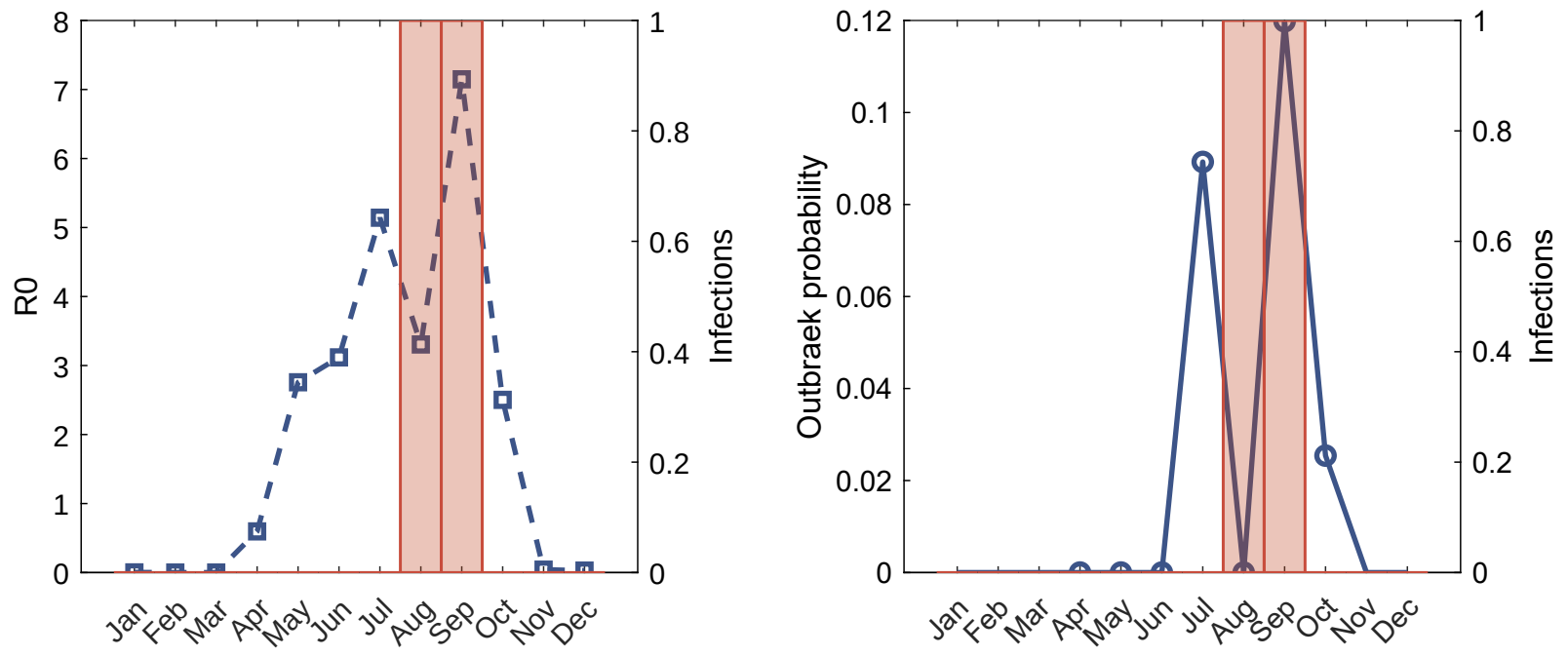

(b) 2016

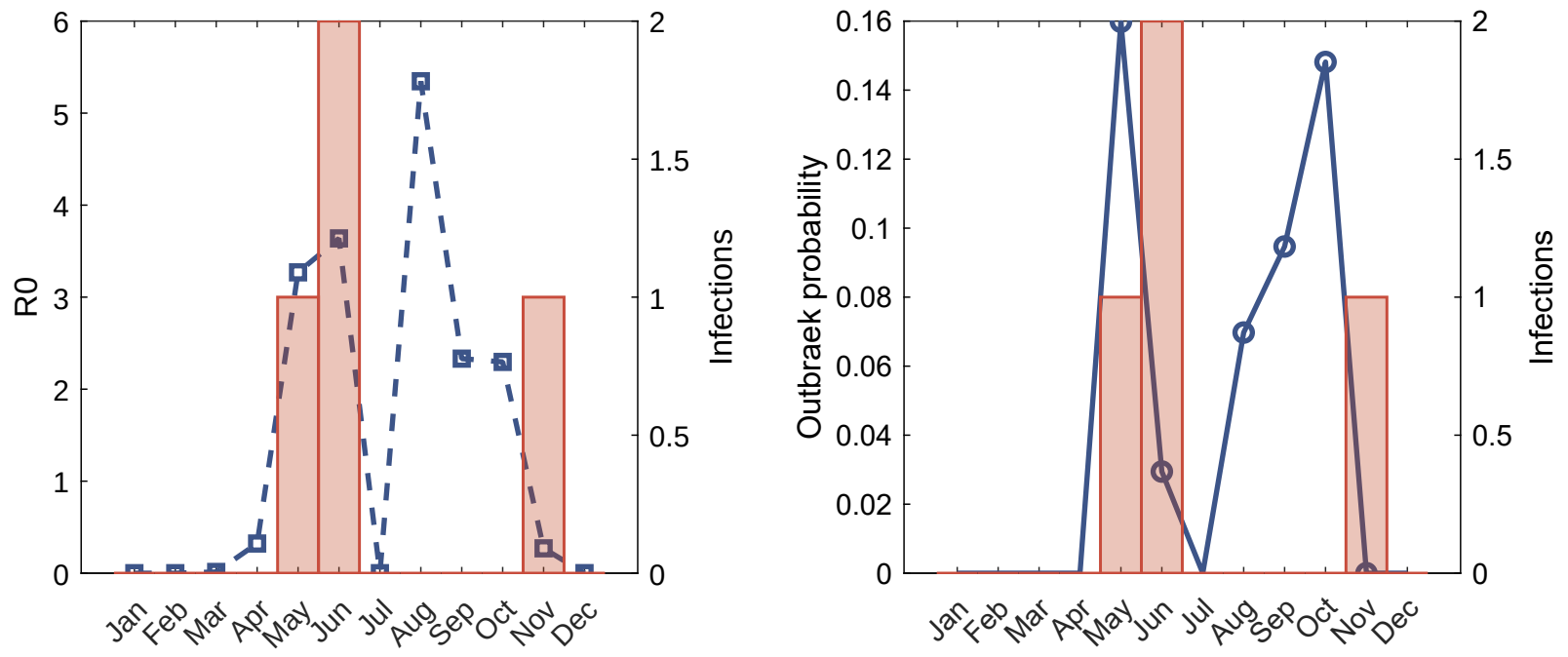

(c) 2017

Fig B: Temporal patterns of R_0 and outbreak probability (2015-2017). The left panels show the basic reproduction number calculated under climate conditions from 2015 to 2017; The right panels show the outbreak probability triggered by imported infections.

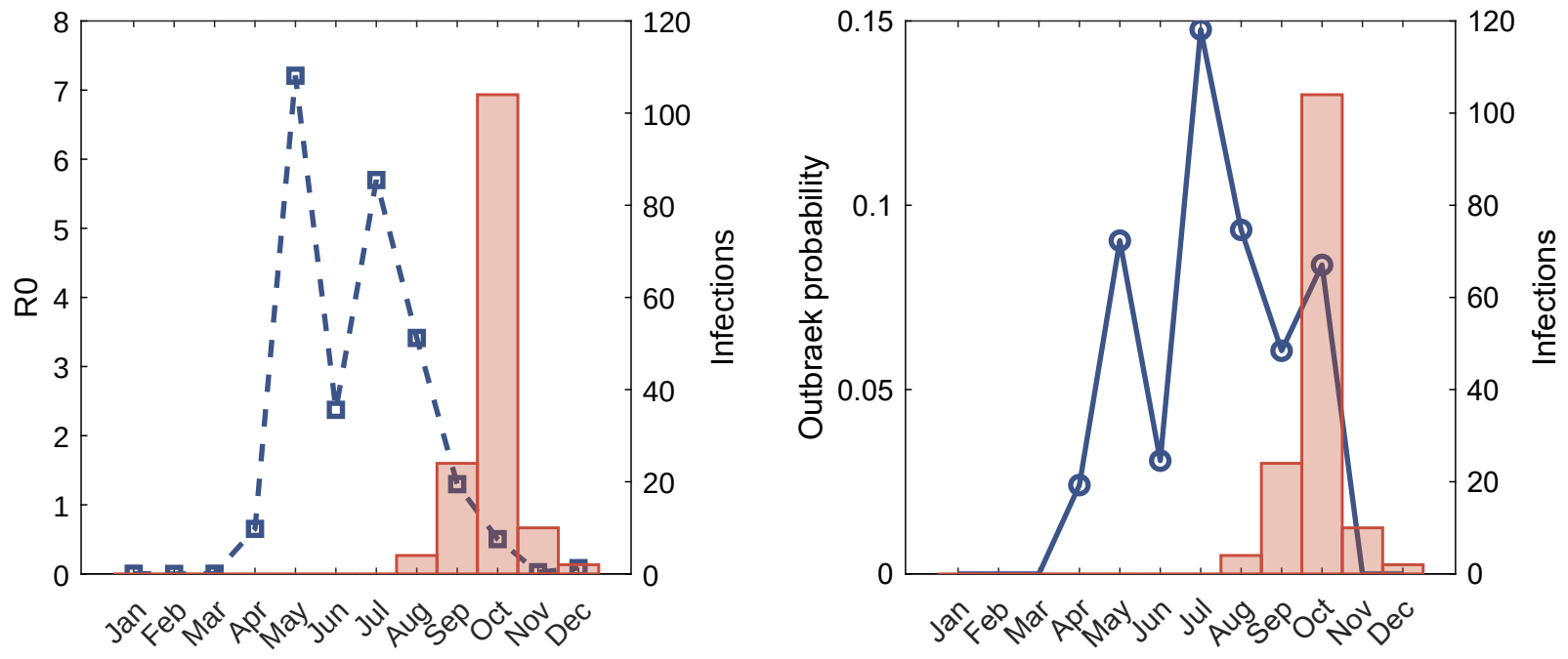

(a) 2018

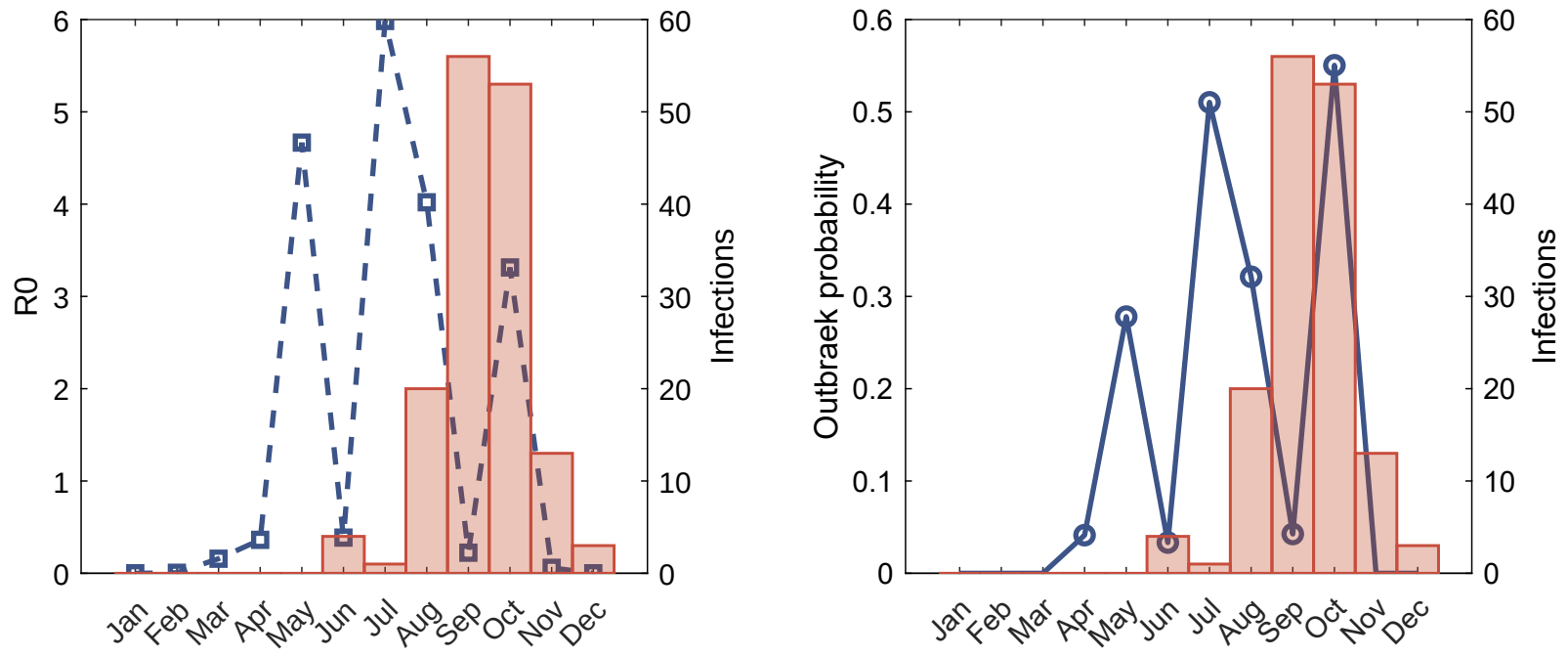

(b) 2019

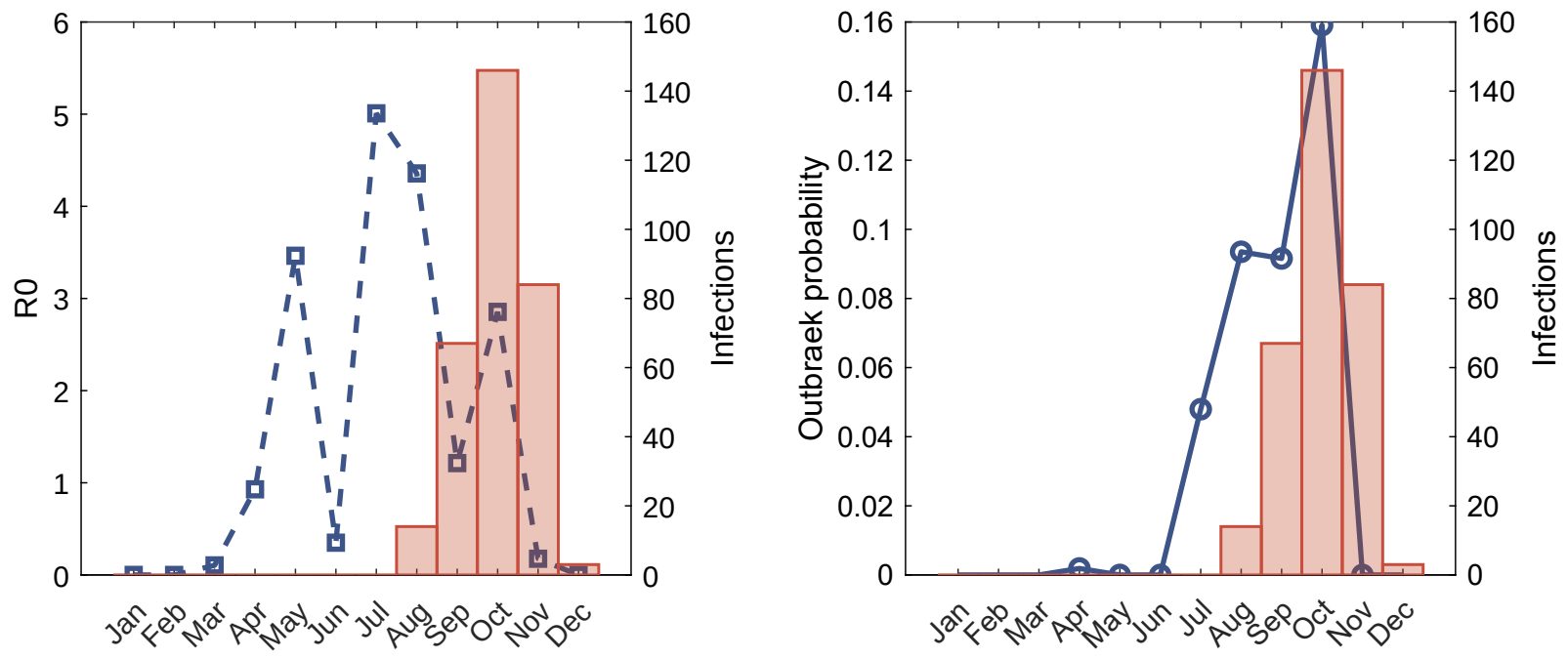

(c) 2023

Fig C: Temporal patterns of R_0 and outbreak probability (2018-2019,2023). The left panels show the basic reproduction number calculated under climate conditions from 2018 to 2023; The right panels show the outbreak probability triggered by imported infections.

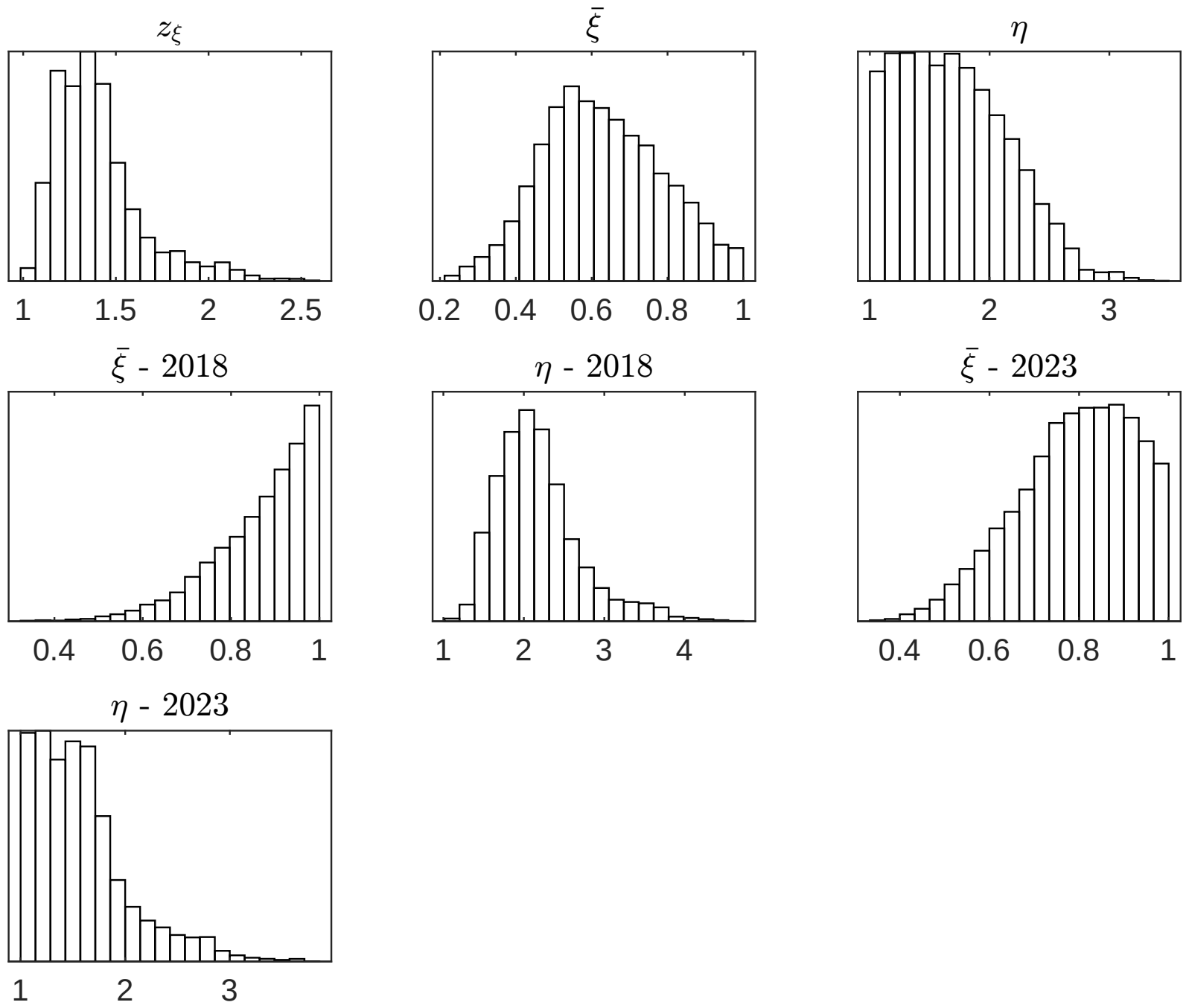

Fig D: Posterior probability density distributions for key epidemiological parameters estimated by MCMC

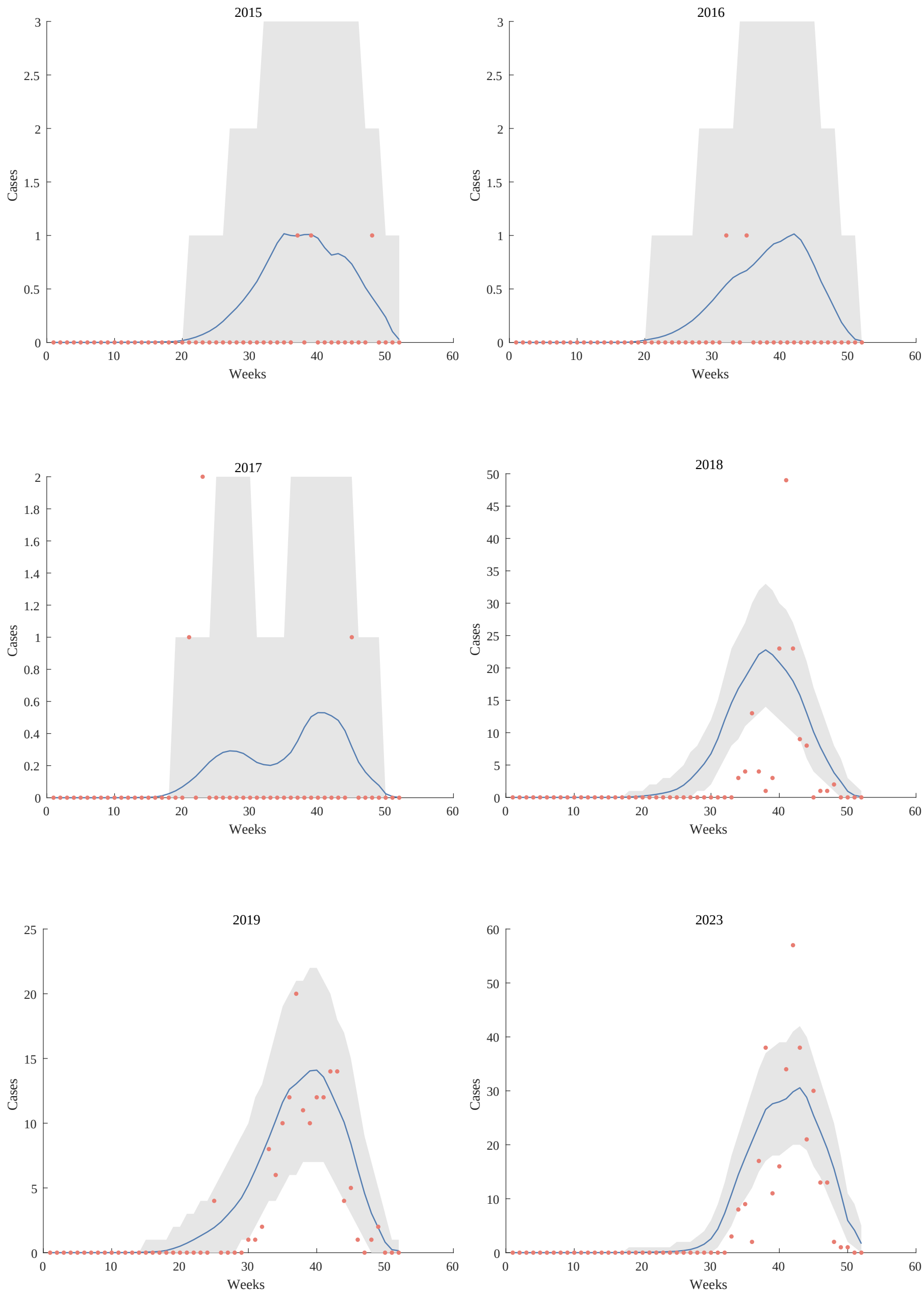

Fig E: Fitting results of the MCMC calibration.

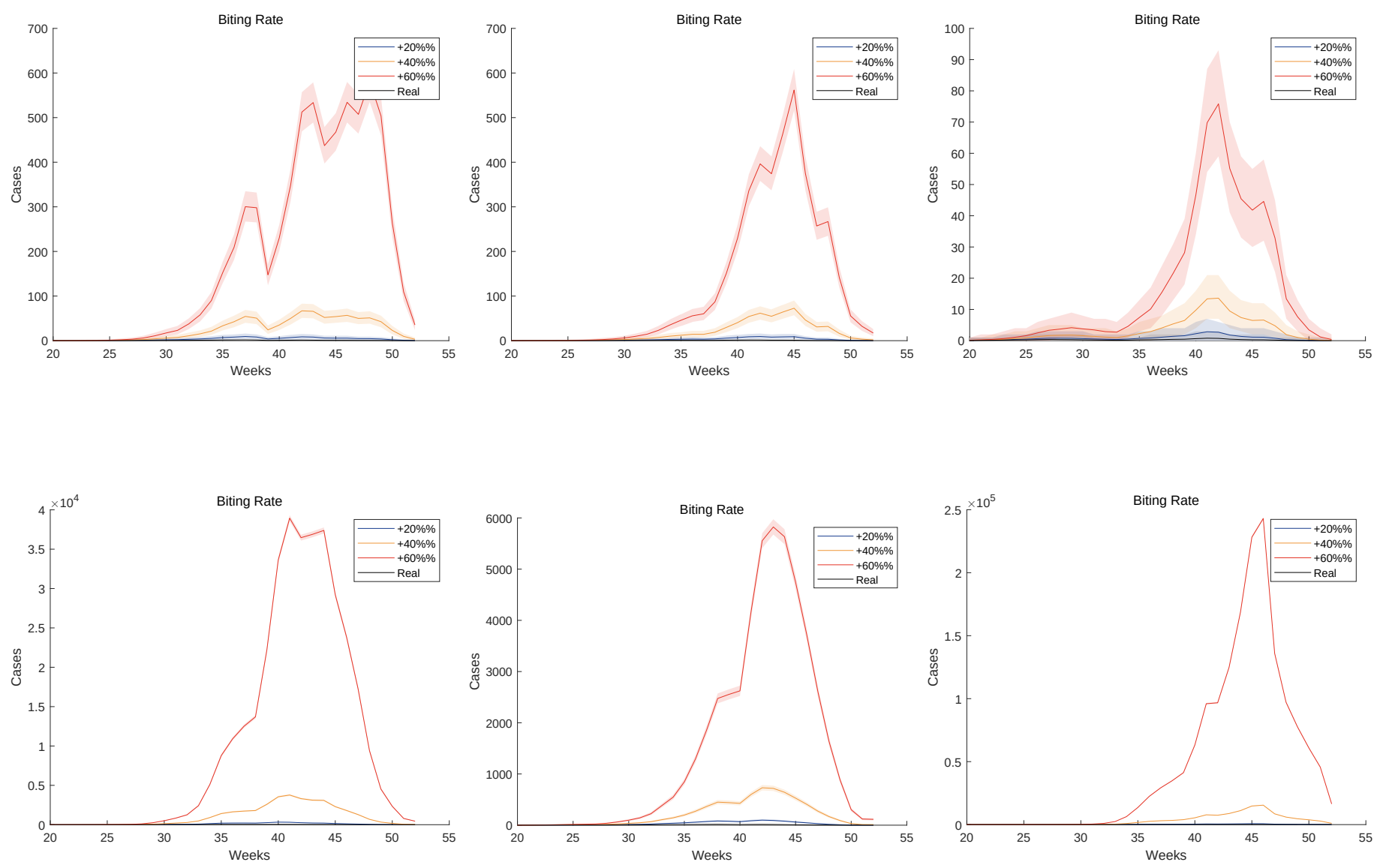

Fig F: Simulated epidemic outcomes under varying biting rates across years.

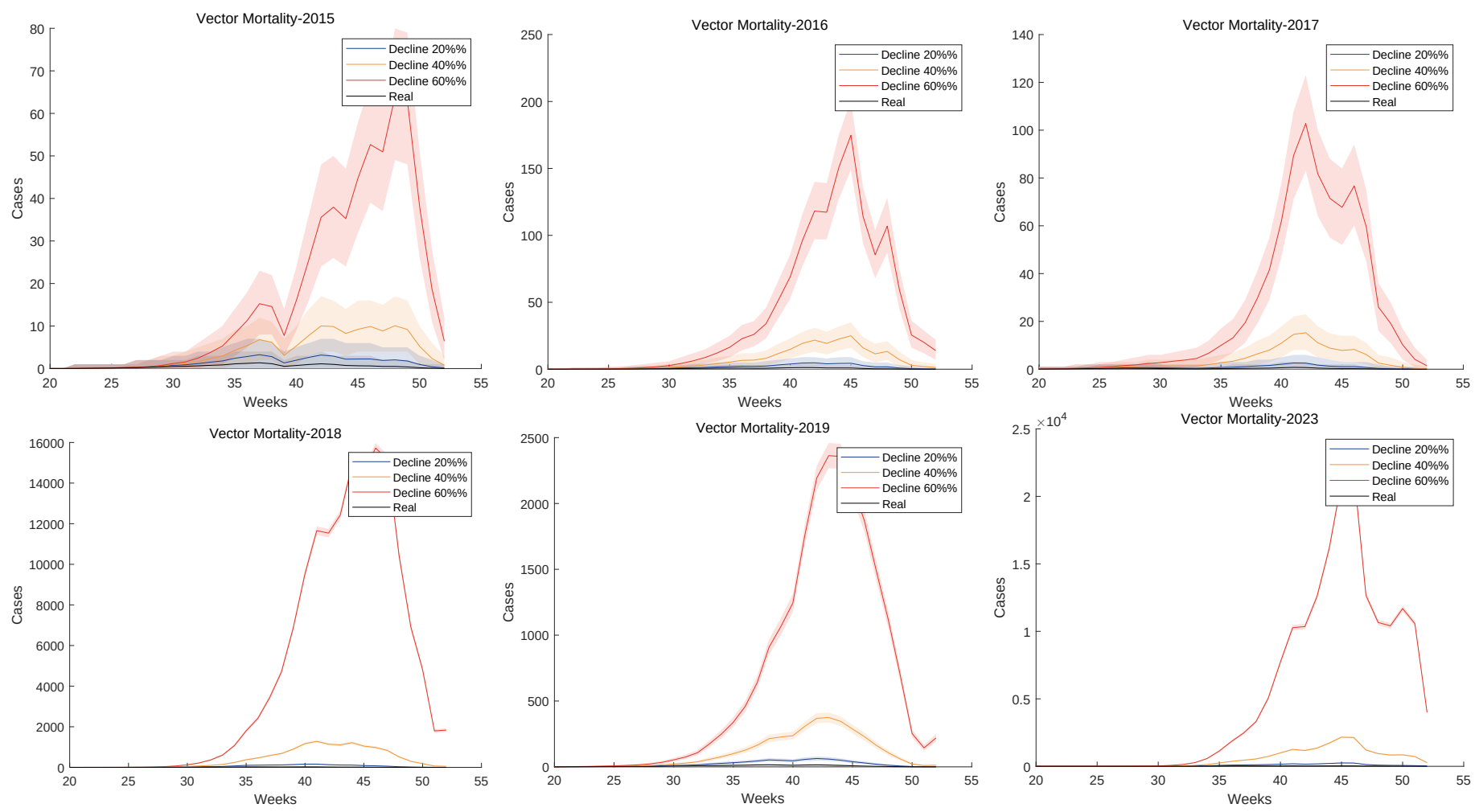

Fig G: Simulated epidemic outcomes under varying vector mortality rates across years.

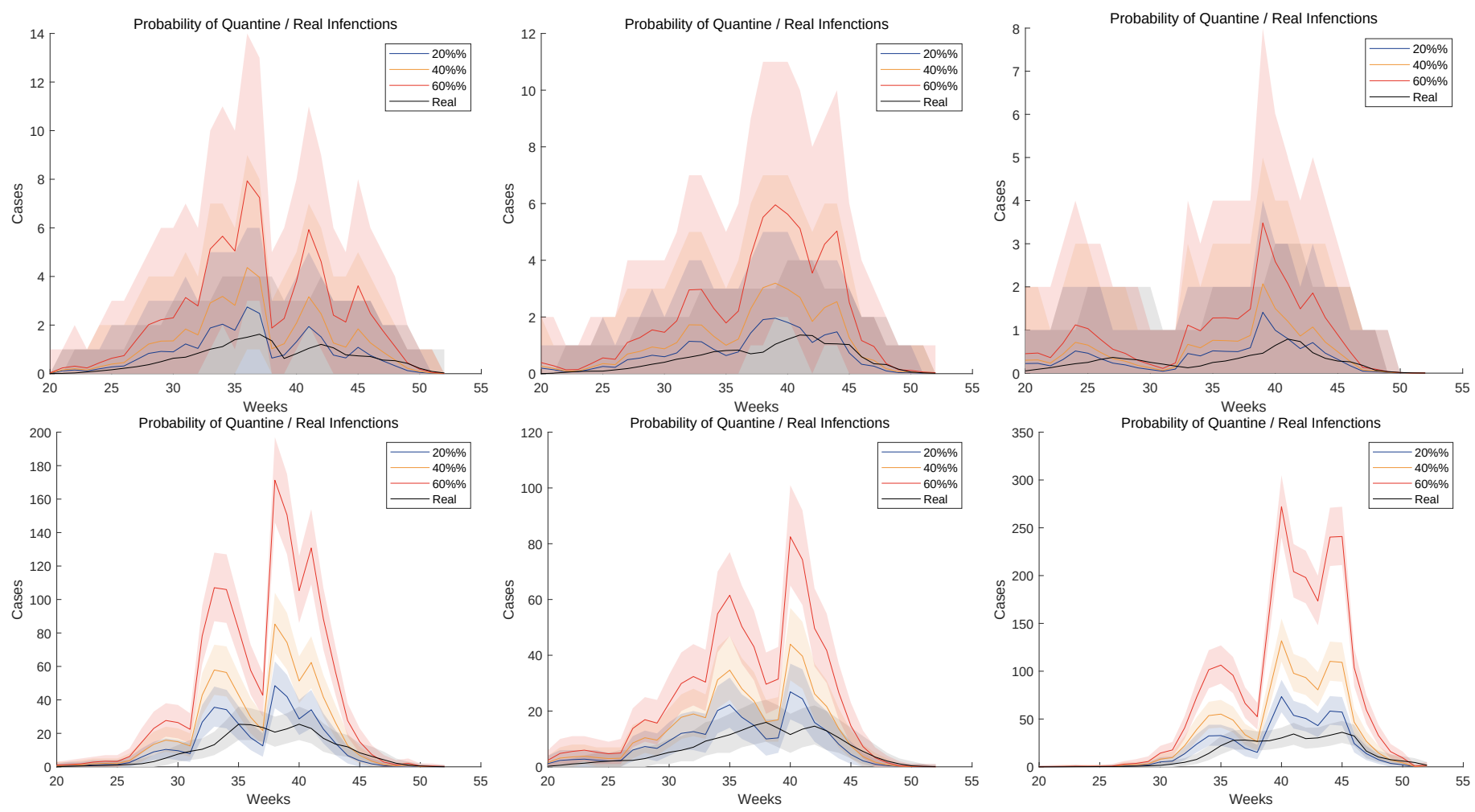

Fig H: Simulated epidemic outcomes under varying quarantine probabilities across years, quantified by total infections rather than reported cases.

Table A: Temperature-dependent functional forms of the key coefficients for vector traits.

**Trait**	**Function**	**c (in 10^{-4})**	**Tmin (°C)**	**Tmax (°C)**
EFD	Briere	85.6	14.58	34.61
pEA	Quadratic	−59.90	13.56	38.29
MDR	Briere	0.79	11.36	39.17
b	Briere	2.02	13.35	40.08
$1/\mu_v$	Quadratic	−1480	9.16	37.73
β_h	Briere	8.49	17.05	35.83
β_v	Briere	4.91	12.22	37.46
γ_v	Briere	0.67	10.68	45.90

*Briere function: $cT(T - T_{\text{min}})(T_{\text{max}} - T)^{1/2}$; Quadratic function: $c(T - T_{\text{max}})(T - T_{\text{min}})$; T means daily mean temperature, T_{max} and T_{min} mean the maximum and minimum temperatures for each trait; c is the rate constant. The parameters were assumed based on Mordecai et al., 2017.

Table B: Monthly sensitivity index of key epidemiological parameters.

Parameter	Year	Average	Jan	Feb	Mar	Apr	May	Jun	Jul	Aug	Sep	Oct	Nov	Dec
Vector mortality	2015	-0.04	0.00	0.00	0.00	0.00	-0.03	0.00	-0.06	0.00	-0.04	-0.33	-0.09	0.00
	2016	-0.04	0.00	0.00	0.00	0.00	-0.03	0.00	-0.06	0.00	-0.04	-0.33	-0.09	0.00
	2017	-0.04	0.00	0.00	0.00	0.00	-0.03	0.00	-0.06	0.00	-0.04	-0.33	-0.09	0.00
	2018	-0.02	0.00	0.00	0.00	0.00	-0.02	0.00	-0.02	0.00	-0.01	-0.13	-0.08	0.00
	2019	-0.04	0.00	0.00	0.00	0.00	-0.03	0.00	-0.06	0.00	-0.04	-0.33	-0.09	0.00
	2023	-0.04	0.00	0.00	0.00	0.00	-0.03	0.00	-0.04	0.00	-0.02	-0.25	-0.10	0.00
Detect rate	2015	-0.02	0.00	0.00	0.00	0.00	-0.01	0.00	-0.02	0.00	-0.01	-0.13	-0.03	0.00
	2016	-0.02	0.00	0.00	0.00	0.00	-0.01	0.00	-0.02	0.00	-0.01	-0.13	-0.03	0.00
	2017	-0.02	0.00	0.00	0.00	0.00	-0.01	0.00	-0.02	0.00	-0.01	-0.13	-0.03	0.00
	2018	-0.01	0.00	0.00	0.00	0.00	-0.01	0.00	-0.01	0.00	-0.01	-0.05	-0.03	0.00
	2019	-0.02	0.00	0.00	0.00	0.00	-0.01	0.00	-0.02	0.00	-0.01	-0.13	-0.03	0.00
	2023	-0.01	0.00	0.00	0.00	0.00	-0.01	0.00	-0.02	0.00	-0.01	-0.10	-0.04	0.00
Prop. of being quarantined	2015	-0.01	0.00	0.00	0.00	0.00	-0.01	0.00	-0.01	0.00	-0.01	-0.07	-0.02	0.00
	2016	-0.01	0.00	0.00	0.00	0.00	-0.01	0.00	-0.01	0.00	-0.01	-0.07	-0.02	0.00
	2017	-0.01	0.00	0.00	0.00	0.00	-0.01	0.00	-0.01	0.00	-0.01	-0.07	-0.02	0.00
	2018	0.00	0.00	0.00	0.00	0.00	0.00	0.00	0.00	0.00	0.00	-0.03	-0.02	0.00
	2019	-0.01	0.00	0.00	0.00	0.00	-0.01	0.00	-0.01	0.00	-0.01	-0.07	-0.02	0.00
	2023	-0.01	0.00	0.00	0.00	0.00	-0.01	0.00	-0.01	0.00	-0.01	-0.05	-0.02	0.00
Quarantine quality	2015	0.00	0.00	0.00	0.00	0.00	0.00	0.00	0.00	0.00	0.00	0.00	0.00	0.00
	2016	0.00	0.00	0.00	0.00	0.00	0.00	0.00	0.00	0.00	0.00	0.00	0.00	0.00
	2017	0.00	0.00	0.00	0.00	0.00	0.00	0.00	0.00	0.00	0.00	0.00	0.00	0.00
	2018	0.00	0.00	0.00	0.00	0.00	0.00	0.00	0.00	0.00	0.00	0.00	0.00	0.00
	2019	0.00	0.00	0.00	0.00	0.00	0.00	0.00	0.00	0.00	0.00	0.00	0.00	0.00
	2023	0.00	0.00	0.00	0.00	0.00	0.00	0.00	0.00	0.00	0.00	0.00	0.00	0.00
Biting rate	2015	0.08	0.00	0.00	0.00	0.00	0.05	0.00	0.10	0.00	0.06	0.56	0.15	0.00
	2016	0.08	0.00	0.00	0.00	0.00	0.05	0.00	0.10	0.00	0.06	0.56	0.15	0.00
	2017	0.08	0.00	0.00	0.00	0.00	0.05	0.00	0.10	0.00	0.06	0.56	0.15	0.00
	2018	0.04	0.00	0.00	0.00	0.00	0.03	0.00	0.04	0.00	0.02	0.23	0.13	0.00
	2019	0.08	0.00	0.00	0.00	0.00	0.05	0.00	0.10	0.00	0.06	0.56	0.15	0.00
	2023	0.06	0.00	0.00	0.00	0.00	0.04	0.00	0.07	0.00	0.04	0.42	0.16	0.00

Table C: Classification of shared and disease-specific parameters for the SEI-SEIQR model. The parameters are divided into distinct categories. Notably, parameters related to the host and vector are transferable between diseases with similar dynamics, such as Dengue and Chikungunya.

Category	Symbol	Definition / Functional Role
**Shared**	μ_n	Human natural birth rate (per 1,000 people)
(Vector & Host)	μ_h	Human natural death rate (per 1,000 people)
	ie	Net immigration/emigration rate
	b	Mosquito biting rate
	EFD	Eggs laid per female mosquito per day
	pEA	Probability of mosquito egg-to-adult survival
	MDR	Mosquito egg-to-adult development rate (per day)
	μ_v	Natural mortality rate of mosquito vector
	K	Environmental carrying capacity for mosquitoes
	z_ξ	Localization coefficient for biting rate adjustment
	ξ	Reduction in biting rate following risk map release
	η	Increase in vector mortality due to control policies
**Disease-specific**	$1/\gamma_h$	Human intrinsic incubation period (days)
(Viral Dynamics)	$1/\sigma_h$	Human infectious period (days)
	$1/\sigma_q$	Detection interval for local infections (days)
	$1/\theta_h$	Duration of quarantine period (days)
	p_q	Probability of case detection
	p_i	Probability of entering quarantine state upon infection
	β_h	Vector-to-human transmission probability per bite
	β_v	Human-to-vector transmission probability per bite
	$1/\gamma_v$	Vector extrinsic incubation period (days)
